# Calmangafodipir Reduces Sensory Alterations and Prevents Intraepidermal Nerve Fibers Loss in a Mouse Model of Oxaliplatin Induced Peripheral Neurotoxicity

**DOI:** 10.3390/antiox9070594

**Published:** 2020-07-07

**Authors:** Annalisa Canta, Alessia Chiorazzi, Eleonora Pozzi, Giulia Fumagalli, Laura Monza, Cristina Meregalli, Valentina A. Carozzi, Virginia Rodriguez-Menendez, Norberto Oggioni, Jacques Näsström, Paola Marmiroli, Guido Cavaletti

**Affiliations:** 1 Experimental Neurology Unit, School of Medicine and Surgery, University of Milano-Bicocca, Via Cadore 48, 20900 Monza, Italy; annalisa.canta@unimib.it (A.C.); alessia.chiorazzi@unimib.it (A.C.); e.pozzi18@campus.unimib.it (E.P.); g.fumagalli14@campus.unimib.it (G.F.); laura.monza@unimib.it (L.M.); cristina.meregalli@unimib.it (C.M.); valentina.carozzi1@unimib.it (V.A.C.); virginia.rodriguez1@unimib.it (V.R.-M.); norberto.oggioni@unimib.it (N.O.); guido.cavaletti@unimib.it (G.C.); 2 PledPharma AB, Grev Turegatan 11 C, 114 46 Stockholm, Sweden; jacques.nasstrom@pledpharma.se; 3 Department of Biotechnology and Biosciences, University of Milano-Bicocca, Piazza della Scienza 1, 20126 Milan, Italy

**Keywords:** calmangafodipir, oxaliplatin, IENF density, cold hyperalgesia, mechanical allodynia, neurotoxicity

## Abstract

Oxaliplatin (OHP) is an antineoplastic compound able to induce peripheral neurotoxicity. Oxidative stress has been suggested to be a key factor in the development of OHP-related peripheral neurotoxicity. Mangafodipir, a contrast agent possessing mitochondrial superoxide dismutase (MnSOD)-mimetic activity, has been tested as a cytoprotector in chemotherapy-induced peripheral neurotoxicity (CIPN). Calmangafodipir (PledOx^®^) has even better therapeutic activity. We investigated a BALB/c mouse model of OHP-related CIPN and the effects of the pre-treatment of calmangafodipir (2.5, 5, or 10 mg/kg intravenously) on sensory perception, and we performed a pathological study on skin biopsies to assess intraepidermal nerve fiber (IENF) density. At the end of the treatments, OHP alone or in pre-treatment with calmangafodipir 2.5 and 10 mg/kg, induced mechanical allodynia and cold thermal hyperalgesia, but calmangafodipir 5 mg/kg prevented these effects. Accordingly, OHP alone or in pre-treatment with calmangafodipir 2.5 and 10 mg/kg, induced a significant reduction in IENF density, but calmangafodipir 5 mg/kg prevented this reduction. These results confirm a protective effect of calmangafodipir against OHP-induced small fiber neuropathy. Interestingly, these results are in agreement with previous observations suggesting a U-shaped effect of calmangafodipir, with the 10 mg/kg dose less effective than the lower doses.

## 1. Introduction

Oxaliplatin (OHP) is an antineoplastic compound commonly used in clinical practice for many types of solid tumors. Despite its proven effectiveness as an anticancer drug, the main side effect observed following chronic administration of OHP is sensory chemotherapy-induced peripheral neurotoxicity (CIPN). In many patients, it is often associated with the appearance of neuropathic pain that compromises their quality of life and frequently leads to modification or discontinuation of normal treatment cycles. There is currently no fully effective drug therapy for peripheral neurotoxicity induced by OHP [1,2]. 

Reactive oxygen (ROS) and nitrogen (RNS) species are known to participate in pathological tissue damage, for instance, during treatment with chemotherapy drugs in cancer patients [3], and the mitochondrial superoxide dismutase (MnSOD) normally keeps ROS and RNS levels under control. Mangafodipir, a contrast agent used in magnetic resonance imaging (MRI), was discovered to possess MnSOD-mimetic [4,5] as well as iron chelating [6] activity and it was tested as a cytoprotector in several contexts, including peripheral neurotoxicity from chemotherapy [7,8]. Mangafodipir has been safely used as a single-dose MRI contrast agent on more than 200,000 patients [9]. However, the difference between the MRI treatment regimen and a potential prevention of CIPN, is that mangafodipir would have to be given repeatedly in conjunction with each chemotherapy cycle, which means a risk of potentially neurotoxic manganese accumulation in the brain. 

To address this shortcoming, another compound derived from mangafodipir, calmangafodipir (proprietary name: PledOx®), was developed, where 80% of the manganese was replaced with calcium. The rationale for this modification is that calcium has a lower affinity for fodipir than manganese [10]. The hypothesis was that zinc in the circulation would preferably displace calcium instead of manganese, which is one factor contributing to an even better therapeutic activity [9,11,12]. 

In fact, calmangafodipir was evaluated in a clinical trial of 173 patients with colorectal cancer treated with OHP-based chemotherapy, and in this phase II, randomized, placebo-controlled, double blind-trial, pre-treatment with PledOx® significantly reduced OHP-induced sensory symptoms without reducing its antineoplastic efficacy [11]. Moreover, it is currently being evaluated in the two phase III POLAR studies [13].

The aim of this study is to reproduce the neuroprotective efficacy of PledOx® using a well-established OHP-induced peripheral neurotoxicity mouse model in order to investigate, at the behavioral and pathological levels, its effects [14].

## 2. Materials and Methods 

### 2.1. Animals and Drugs

The study was approved by the Italian Ministry of Health (approval number: 169/2019-PR 25/2/2019), in agreement to all guidelines set forth in the Guide for the Care and Use of Laboratory Animals (U.S. National Research Council), as well as to the Italian D.L.vo n. 26/2014 in compliance with the European Union directive 2010/63/UE, and by the University of Milano-Bicocca Ethics Committee (protocol number 0040933/19, May 27, 2019). The animals were housed in a limited-access and certified facility (temperature 21 ± 2 °C, humidity 50% ± 20%) and there was an artificial 12:12 h light cycle from 7:00 a.m. to 7:00 p.m. 

Animal health status was assessed daily, and body weight was recorded twice a week during the treatment period to monitor general conditions and to adjust the dose, and once a week during a follow-up period. Mice were housed 2/3 in Makrolon (Tecniplast, Gazzada, Italy) cages, fed with Global Diet 2018 pellets (Mucedola srl, Settimo Milanese, Italy) and purified drinking water ad libitum.

OHP (5% glucose liquid solution, Accord Healthcare, Limited, Middlesex, UK) was delivered intravenously (i.v.) via the tail vein using a 10 mL/kg volume of administration and used immediately after dilution 1:10 of 50 mg/10 mL stock solution. Calmangafodipir powder (PledOx^®^, batch number M190000030, AMRI’s batch number 12417, PledPharma AB, Stockholm, Sweden) was dissolved in sterile saline solution at a dose of 10 mg/kg (stock solution), divided in vials, and frozen until use. The other solutions (5 and 2.5 mg/kg) were obtained by dilution in sterile saline of the stock and used immediately after reconstruction. Calmangafodipir was i.v.-administered slowly 10 min before OHP injection using a 10 mL/kg volume of administration.

### 2.2. Study Design

**Figure 1:**
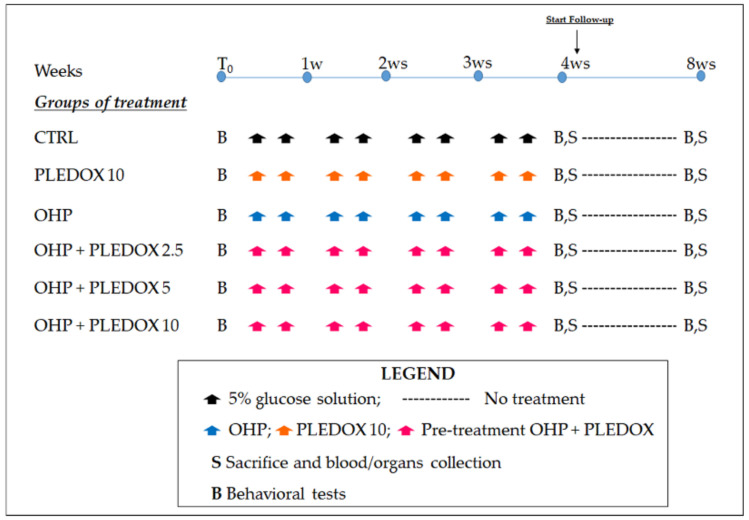
Flow-chart of the study. In the pre-treatment oxaliplatin (OHP) + PLEDOX groups, PLEDOX was administered 10 min before OHP.

A total of 120 male BALB/c mice (10 weeks of age, Envigo, Bresso, Italy) were used for the study. They were divided into 6 groups of 20 mice each. Half of the animals belonging to each group were sacrificed at the end of the treatment period by supramaximal anesthesia, while the remaining mice were observed for a follow-up period of 4 weeks and then sacrificed. The 6 groups of animals were treated as follows: 5% glucose solution (CTRL), calmangafodipir 10 mg/kg twice a week for 4 weeks (PLEDOX 10), OHP 5 mg/kg twice a week for 4 weeks (OHP), OHP and calmangafodipir 2.5 mg/kg pre-treatment twice a week for 4 weeks (OHP + PLEDOX 2.5), OHP and calmangafodipir 5 mg/kg pre-treatment twice a week for 4 weeks (OHP + PLEDOX 5), OHP and calmangafodipir 10 mg/kg pre-treatment twice a week for 4 weeks (OHP + PLEDOX 10). Animals were randomized into each group based on pre-treatment assessment of behavioral tests to ensure comparable baseline conditions. At baseline, at the end of treatment and after a 4-week follow-up period, all mice were tested for thermal (cold) and mechanical thresholds. At sacrifice (end of treatment and end of the follow-up period), blood, caudal nerve, and hind-paw skin specimens were collected from all groups of mice. The flow-chart of the study is represented in Figure 1.

### 2.3. Behavioral Tests

#### 2.3.1. Dynamic Aesthesiometer Test

A Dynamic Aesthesiometer Test (model 37450, Ugo Basile Biological Instruments, Comerio, Italy), which produced a mechanical force that increased linearly, was used to assess the development of mechanical allodynia.

At each time of detection and after acclimatization, a 0.5 mm diameter pointed metallic filament was positioned under the surface of the animal hind-paw. This mechanical stimulus started to exert a gradually growing punctuating pressure that reached 15 gr within 15 s and evoked an evident voluntary hind-paw withdrawal reaction that was recorded automatically and represented the mechanical nociceptive threshold. This evaluation was performed three times for each hind-paw to yield a mean value. An upper limit cut-off of 15 s was established, after which the mechanical stimulus was automatically switched off [15].

#### 2.3.2. Cold Plate Test 

The cold nociceptive threshold was assessed using the Cold Plate Test (model 35100 - Hot/Cold Plate, Ugo Basile) that was composed of a cylinder made in Plexiglas and a thermostatic plate that generated a variable temperature.

Mice, which were free to walk and move, were positioned on the plate fixed at 4 °C, and the number of pain signs clearly different from normal behavior observed by a trained examiner (EP) (e.g., abnormal modifications in rear and tail movements, sudden jumping, shaking, and rear licking) was documented in a 5 min trial.

As previously described, and according to a predefined severity checklist, if mice evidenced severe intolerance to temperature (namely suggested by vocalization and clearly anxious behavior), the trial was prematurely interrupted [15].

### 2.4. Sampling and Processing of Organs/Tissues

At the end of treatment and at the end of the follow-up period, animals were sacrificed. At each time point, caudal nerves were obtained for morphological analysis from 4 animals/group, while skin biopsies were collected for morphometric examination of intraepidermal nerve fibers (IENF). Moreover, at each time point, blood samples were collected for blood cell counts from 6 mice/group, in order to test the hematological toxicity of OHP and to confirm that the schedule selected for the study was able to generate a non-neurological picture similar to those observed in clinical practice. For caudal nerve processing, a previously published method was followed [16,17].

The caudal nerves were collected and put in 3% glutaraldehyde at room temperature for 3 h. The specimens were then post-fixed in OsO_4_ and embedded in epoxy resin. Morphological analysis was performed on 1 μm-thick methylene blue stained semithin sections using a Nikon Eclipse E200 light microscope (Leica Microsystems GmbH, Wetzlar, Germany). For IENF density assessment, a previously published method was followed [18]. Punch biopsy (3 mm) from one hind-paw of each animal was collected after sacrifice. Samples were fixed in 2% paraformaldehyde-lysine-periodate sodium solution. Morphometrical analysis was carried out on 20 μm-thick sections. For each footpad, three sections were randomly selected and immunostained with rabbit polyclonal antiprotein gene product 9.5 (PGP 9.5; GeneTex, Irvine, CA, USA). Under a light microscope (Nikon Eclipse E200 light microscope, Leica Microsystems GmbH, Wetzlar, Germany) at 40× magnification, the total number of PGP-positive fibers was counted. Only the fibers that crossed the dermal–epidermal junction were counted, and secondary branching was excluded. After the measurement of epidermidis length, the linear density of IENF/mm was calculated. All the analyses were performed in a blind fashion by the same examiner.

### 2.5. Data Acquisition

Whenever possible, online software packages specifically aimed for the test’s purposes were used for recording the data for all investigations. If the online registration was not possible, the raw data were handwritten and subsequently entered manually into the computer and archived at the School of Medicine and Surgery, University of Milano-Bicocca.

### 2.6. Statistical Evaluation

The differences in body weights, behavioral tests, and IENF density evaluations were statistically analyzed using a 2-step approach with the nonparametric one-way ANOVA test Kruskall-Wallis, followed by Dunn post-test (significance level set at *p* < 0.05). The blood cell count was performed only for exploratory aims and the study was not powered to allow a statistical comparison among groups for these results.

## 3. Results

### 3.1. Clinical Observations and Body Weight Changes

**Figure 2:**
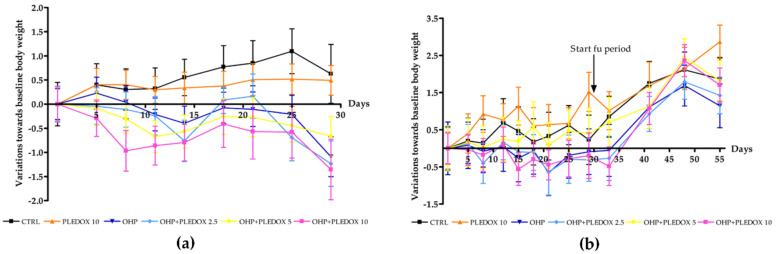
Body weight changes during the study (**a**): mice that performed only 4 weeks of treatment; (**b**): mice that also completed 4 weeks of a follow-up period, mean ± SD).

The administration of OHP and PLEDOX alone or in pre-treatment was generally well tolerated. Body weight changes along the study (Figure 2a,b) confirmed the good tolerability of OHP and PLEDOX alone or in pre-treatment, with a mild reduction in body weight only in the OHP + PLEDOX 10 group.

### 3.2. Dynamic Aesthesiometer Test

**Figure 3:**
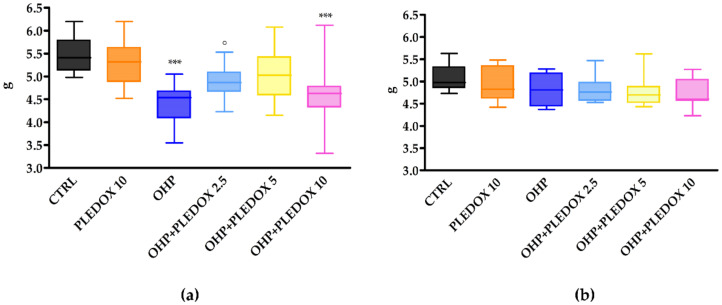
Dynamic Aesthesiometer Test at the end of treatment (**a**), and at the end of the follow-up period (**b**). *** *p* < 0.001 vs. CTRL, ○ *p* < 0.05 vs. CTRL, Kruskal-Wallis test.

The Dynamic Aesthesiometer Test was evaluated at baseline, at the end of treatment, and after 4 weeks of follow-up. At baseline, there was no statistically significant difference among groups (data not shown). At the end of treatment (Figure 3a), mice treated with OHP alone or with PLEDOX 2.5 and 10 mg/kg pre-treatment showed the development of mechanical allodynia (OHP *p* < 0.001 vs. CTRL; OHP + PLEDOX 2.5 *p* < 0.05 vs. CTRL and OHP + PLEDOX 10 *p* < 0.001 vs. CTRL), while this was not observed in the OHP + PLEDOX 5 group. At the end of the follow-up period (Figure 3b), no statistically significant differences were observed among groups. 

### 3.3. Cold Plate Test 

**Figure 4:**
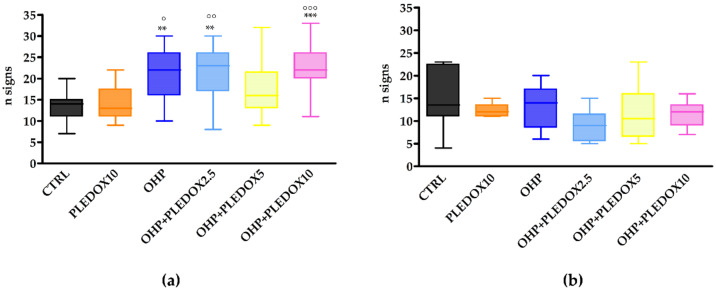
Cold Plate Test at the end of treatment (**a**), and at the end of the follow-up period (**b**). ° *p* < 0.05 vs. PLEDOX, °° *p* < 0.01 vs. PLEDOX, °°° *p* < 0.001 vs. PLEDOX, ** *p* < 0.01 vs. CTRL, *** *p* < 0.001 vs. CTRL, Kruskal-Wallis test.

The Cold Plate Test was evaluated at baseline, at the end of treatment, and after 4 weeks of the follow-up period. At baseline, there was no statistically significant difference among groups (data not shown). At the end of treatment (Figure 4a), mice treated with OHP alone or with PLEDOX 2.5 and 10 mg/kg pre-treatment showed the development of cold hyperalgesia (OHP and OHP + PLEDOX 2.5 *p* < 0.01 vs. CTRL; OHP + PLEDOX 10 *p* < 0.001 vs. CTRL), while this was not observed in the OHP + PLEDOX 5 group. At the end of the follow-up period (Figure 4b), no statistically significant differences were observed among groups. 

### 3.4. Blood Cell Count

At the end of treatment and at the end of the follow-up period, blood was collected for blood cell counts from 6 mice/group. Chronic treatment with OHP induced marked anemia, leukocytopenia, and thrombocytopenia at the end of the treatment period. The pre-administration of any PLEDOX dose was not able to prevent these changes, apart from a nearly complete prevention of OHP-induced effects on platelet counts observed in the animals pre-treated with the doses of 5 and 10 mg/kg (Table 1). At the end of the follow-up period, all the abnormal values (except OHP + PLEDOX 5) tended to return close to control values (Table 2). 

### 3.5. Caudal Nerve Morphological Analysis

**Figure 5:**
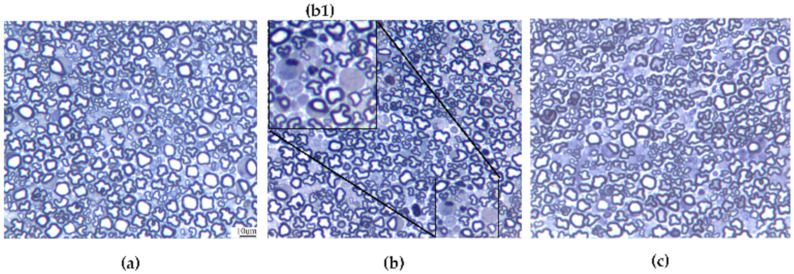
Representative images of caudal nerve samples from a control (**a**), OHP-treated mice (**b**), and OHP + PLEDOX 5-treated mice (**c**) at the end of treatment. (**b1** represents a magnification showing degenerating axons) (bar = 10 μm, insert b1 = X4 magnification).

At the end of treatment and at the end of the follow-up period, caudal nerves were collected from 4 mice/group and morphological analysis was performed. Mild axonopathy was observed in all the OHP-treated mice, including those pre-treated with PLEDOX at any dose. Representative caudal nerve images showing the effect of OHP on nerve fibers are reported in Figure 5.

### 3.6. IENF Density Evaluation

**Figure 6:**
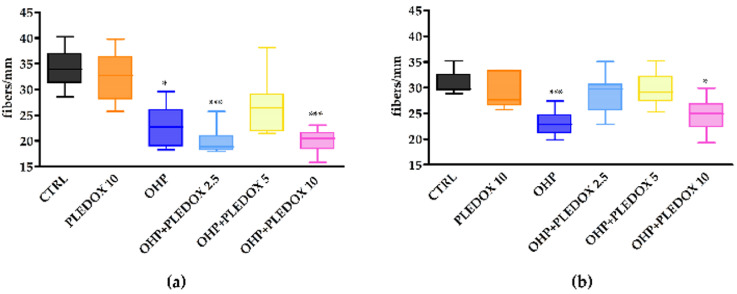
IENF density evaluated at the end of treatment (**a**), and at the end of the follow-up period (**b**). * *p* < 0.05 vs. CTRL, *** *p* < 0.001 vs. CTRL; Kruskal-Wallis test.

At the end of treatment and at the end of the follow-up, skin biopsies were obtained from 4 mice/group and IENF density was evaluated. At the end of treatment (Figure 6a), mice treated with OHP alone or with PLEDOX 2.5 and 10 mg/kg pre-treatment showed a statistically significant reduction of IENF density (OHP *p* < 0.05 vs. CTRL; OHP + PLEDOX 2.5 and OHP + PLEDOX 10 *p* < 0.001 vs. CTRL). This reduction was not present in the OHP + PLEDOX 5 group. At the end of the follow-up period (Figure 6b), the reduction of IENF density was still present only in the OHP and OHP + PLEDOX 10 treated group (OHP *p* < 0.001 vs. CTRL; OHP + PLEDOX 10 *p* < 0.05 vs. CTRL).

## 4. Discussion

Effective prevention of OHP-induced CIPN is still an unmet clinical need, despite extensive efforts at the preclinical and clinical levels. This unsolved issue has relevant clinical implications since it determines treatment changes and long-term impairments with a severe impact on cancer survivors’ quality of life [19,20,21,22]. Among the several reasons limiting an effective approach to OHP-induced neurotoxicity, a major issue, are the incomplete knowledge of its pathogenesis and the use of preclinical assessment methods that are too far from the clinical practice.

One of the mechanisms that seems to be more strongly related to OHP-induced neurotoxicity is oxidative stress, secondary to mitochondrial damage and energy failure, in the context of highly energy-demanding cells such as primary sensory neurons of the dorsal root ganglia (DRG), thus suggesting that prevention of oxidative stress might have a neuroprotective effect [11,19,23,24,25].

The current study demonstrates that the iron chelator and SOD-mimetic drug calmangafodipir can prevent typical manifestations of OHP-induced neurotoxicity in a well-established mouse model, where hematological tests confirmed the similarity with the clinical condition of the selected treatment schedule. Moreover, in this study, the evaluation of calmangafodipir’s effect is not limited to behavioral tests, since it is complemented by the assessment of IENF density, a diagnostic tool for small fiber sensory neuropathy that has been suggested to be an early sign of CIPN [3]. In our study, this assessment allows the pathological confirmation of the behavioral test results, providing them with a strong and reliable support, but it is also important since IENF density measurement is used in the clinical setting, making the translation of the preclinical results much easier and more reliable [26]. Furthermore, there are examples of clinical treatment effects with pharmacological agents that correlate with the reversal of IENF reduction [27]. 

Our results from the blind and objective analysis of IENF density parallel behavioral test results and, interestingly, but expectedly, the dose-response for the treatment effect of calmangafodipir on both the behavioral and IENF density demonstrate a clear U- or bell-shape. 

As described by Cuzzocrea et al. [28] and more recently by Bonetta [29], it is well known that the SOD enzyme and certain SOD-mimetics produce bell-shaped dose response curves. However, the mechanism behind the bell-shaped dose response curves has neither been well understood nor well described in the literature, with a few exceptions. McCord proposed that the mechanism behind the hormesis at the basis of the reduced efficacy with increased SOD concentrations is due to the fact that the superoxide radical may be involved in the increased lipid peroxidation acting both as a creator and a terminator of the free-radical mediated chain reaction [30]. Mao et al. [31] argued that it is the hydroxyl radical and iron that produce the bell-shaped dose response. Both of these articles resonate well with calmangafodipir’s dual mechanism of action as both a SOD-mimetic and iron chelator. However, the results presented in the US patent from 2000 by Towart et al. about various fodipir (DPDP) compounds and the iron chelator dexrazoxane point to the hydroxyl radical and iron as the mechanisms behind the U-shaped dose-response [32]. The results by Towart et al. demonstrate that the iron chelators MnDPDP, DPDP, ZnDPDP, as well as dexrazoxane, all have bell-shaped dose response curves, while this was not seen with MnCl_2_ (which can function as a SOD-mimetic in phosphate buffers in vitro [33]). Since none of DPDP, ZnDPDP, or dexrazoxane are SOD-mimetics, this argues against the superoxide radical as the mediator for the hormesis, and lends support to a mechanism based on iron chelation, i.e., reduction of the Fenton reaction with subsequent reduction in the generation of the hydroxyl radical. However, irrespective of if it is the superoxide radical or the hydroxyl radical that can serve both as an initiator and a terminator of the free radical-mediated chain reaction and that is behind the bell-shaped dose-response, the dual mechanism of blocking both the superoxide-generating pathway as well as the hydroxyl-generating pathway by calmangafodipir, will most likely contribute to the beneficial therapeutic profile of calmangafodipir [9,12].

The effect of calmangafodipir on the platelet count also displayed a tendency for a bell-shaped dose-response. With regards to the effect of calmangafodipir on the other hematological parameters, it differs from an earlier study [12] where it was not only found to protect platelets but also white blood cells in general, and particularly neutrophils. The difference between these two animal studies may reside in the OHP schedules. In fact, the former consisted of a single high-dose OHP administration, while the current one is based on repeated dosing of moderate doses of OHP, in line with the results of the PLIANT study [11]. 

## 5. Conclusions

In conclusion, the current study lends mechanistic support and insights to the findings that the iron chelator and SOD-mimetic calmangafodipir and the related mangafodipir have demonstrated clinical efficacy against OHP-induced CIPN [8,11]. Calmangafodipir’s protective effect was evident at the behavioral level and confirmed by pathological assessment. Despite these, results should be considered as preliminary data with the need for further exploring the redox status of the animals, as they might be relevant not only to neuroprotection, but potentially also to anticancer treatment efficacy. In fact, both the Coriat study [8] and the very first case study by Yri et al. [34] indicate that patients treated with this class of compounds can tolerate very high cumulative doses of OHP without developing severe neurotoxicity, and this might match the observation that in the large SCOT study there was a tendency, albeit far from statistically significant, that increased doses of OHP with FOLFOX gave a better treatment effect [35]. 

## Figures and Tables

**Table 1:** Blood cell counts at the end of treatment.

**Group**	**WBC**	**RBC**	**Hgb**	**Hct**	**MCV**	**MCH**
CTRL	6.2 ± 1.7	9.5 ± 1	15.6 ± 1.8	53.2 ± 6	56.3 ± 0.8	16.4 ± 0.2
PLEDOX 10	8.1 ± 4.1	8.8 ± 1.1	14.5 ± 1.7	49.4 ± 6.6	55.8 ± 1.2	16.5 ± 0.4
OHP	2.5 ± 0.8	4.7 ± 0.7	7 ± 1.4	24.3 ± 4.6	51 ± 2.2	14.6 ± 0.8
OHP + PLEDOX 2.5	2.5 ± 1	4.8 ± 0.3	7.1 ± 0.5	24.5 ± 2.1	51.2 ± 1.3	14.8 ± 0.6
OHP + PLEDOX 5	3.4 ± 0.9	5.1 ± 0.5	8 ± 1.1	27.9 ± 3.9	54.3 ± 2.6	15.5 ± 0.7
OHP + PLEDOX 10	3.2 ± 1	5.2 ± 1	8 ± 2	27.7 ± 7	52.5 ± 3.1	15.1 ± 0.9
**Group**	**MCHC**	**RDW**	**Plt**	**MPV**	**Pct**	**PDW**
CTRL	29.3 ± 0.3	11.9 ± 0.1	566.7 ± 211.2	10.3 ± 0.8	0.6 ± 0.2	12.1 ± 1.9
PLEDOX 10	29.5 ± 0.8	11.7 ± 0.2	507.3 ± 311	10.5 ± 1.1	0.5 ± 0.3	10.6 ± 5.5
OHP	28.7 ± 0.7	12.2 ± 0.5	324 ± 152.7	10.7 ± 0.3	0.3 ± 0.2	15.6 ± 0.4
OHP + PLEDOX 2.5	28.9 ± 1	12.6 ± 0.4	379.5 ± 107.3	10.5 ± 0.3	0.4 ± 0.1	15.7 ± 0.1
OHP + PLEDOX 5	28.5 ± 0.2	13 ± 1.1	496.5 ± 138.2	10.4 ± 0.4	0.5 ± 0.1	15.1 ± 0.7
OHP + PLEDOX 10	28.8 ± 0.4	13.1 ± 1.8	448.8 ± 148	10.4 ± 0.5	0.5 ± 0.1	15.8 ± 0.3

Values represent mean ± SD. WBC = white blood cells, RBC = red blood cells, Hgb = hemoglobin, Hct = hematocrit, MCV = mean corpuscular volume, MCH = mean corpuscular hemoglobin, MCHC = mean corpuscolar hemoglobin concentration, RDW = red blood cells distribution width, Plt = platelets, MPV = mean platelets volume, Pct = platelets hematocrit, PDW = platelet distribution width.

**Table 2:** Blood cell counts at the end of the follow-up period.

**Group**	**WBC**	**RBC**	**Hgb**	**Hct**	**MCV**	**MCH**
CTRL	7.5 ± 2.3	10.1 ± 1.6	17.1 ± 3.3	55.3 ± 9.9	54.5 ± 1.4	16.8 ± 0.6
PLEDOX 10	5.2 ± 2.7	9.2 ± 0.4	15.2 ± 0.7	49.4 ± 2.3	53.7 ± 1	16.4 ± 0.3
OHP	5.3 ± 1.4	8.5 ± 0.9	13.7 ± 1.7	46.1 ± 5.3	54.5 ± 1	16.2 ± 0.5
OHP + PLEDOX 2.5	5.2 ± 1.4	8.7 ± 0.9	14.5 ± 1.9	47.6 ± 5.6	54.3 ± 1	16.6 ± 0.4
OHP + PLEDOX 5	5.2 ± 1.3	7.2 ± 1.9	11.6 ± 3.7	38.9 ± 11.2	54 ± 1.4	15.9 ± 0.9
OHP + PLEDOX 10	5.5 ± 2	8.6 ± 0.3	14.2 ± 0.6	46.9 ± 1.9	54.5 ± 0.5	16.5 ± 0.2
**Group**	**MCHC**	**RDW**	**Plt**	**MPV**	**Pct**	**PDW**
CTRL	30.7 ± 0.5	13.6 ± 0.6	684.2 ± 358.3	10.2 ± 1.4	0.6 ± 0.3	7.1 ± 5.5
PLEDOX 10	30.6 ± 0.2	13.8 ± 0.6	605.7 ± 277.8	9.4 ± 0.4	0.6 ± 0.3	11.9 ± 1.9
OHP	29.8 ± 0.5	13.2 ± 0.4	492.7 ± 239.6	10.5 ± 1	0.5 ± 0.2	11.3 ± 6
OHP + PLEDOX 2.5	30.4 ± 0.5	13.3 ± 0.6	469 ± 176.5	10.4 ± 0.7	0.5 ± 0.2	14.8 ± 1.7
OHP + PLEDOX 5	29.5 ± 1	13.3 ± 1	316 ± 203.5	10.4 ± 0.8	0.3 ± 0.2	12.6 ± 6.5
OHP + PLEDOX 10	30.3 ± 0.2	13.2 ± 0.5	487.2 ± 194.4	10.2 ± 0.4	0.5 ± 0.2	14.4 ± 2.4

## References

[1] Hershman D.L., Lacchetti C., Dworkin R.H., Lavoie Smith E.M., Bleeker J., Cavaletti G., Chauhan C., Gavin P., Lavino A., Lustberg M.B. (2014). Prevention and management of chemotherapy-induced peripheral neuropathy in survivors of adult cancers: American society of clinical oncology clinical practice guideline. J. Clin. Oncol..

[2] Albers J.W., Chaudhry V., Cavaletti G., Donehower R.C. (2014). Interventions for preventing neuropathy caused by cisplatin and related compounds. Cochrane Database Syst. Rev..

[3] Bennett G.J., Doyle T., Salvemini D. (2014). Mitotoxicity in distal symmetrical sensory peripheral neuropathies. Nat. Rev. Neurol..

[4] Asplund A., Grant D., Karlsson J.O. (1994). Mangafodipir (MnDPDP)-and MnCl2-induced endothelium-dependent relaxation in bovine mesenteric arteries. J. Pharmacol. Exp. Ther..

[5] Brurok H., Ardenkjær-Larsen J.H., Hansson G., Skarra S., Berg K., Karlsson J.O., Laursen I., Jynge P. (1999). Manganese dipyridoxyl diphosphate: MRI contrast agent with antioxidative and cardioprotective properties?. Biochem. Biophys. Res. Commun..

[6] Rocklage S.M., Cacheris W.P., Quay S.C., Ekkehardt Hahn F., Raymond K.N. (1989). Manganese(II) N,N′-dipyridoxylethylenediamine-N,N′-diacetate 5,5′-bis(phosphate). Synthesis and characterization of a paramagnetic chelate for magnetic resonance imaging enhancement. Inorg. Chem..

[7] Bedda S., Laurent A., Conti F., Chéreau C., Tran A., Tran-Van Nhieu J., Jaffray P., Soubrane O., Goulvestre C., Calmus Y. (2003). Mangafodipir prevents liver injury induced by acetaminophen in the mouse. J. Hepatol..

[8] Coriat R., Alexandre J., Nicco C., Quinquis L., Benoit E., Chéreau C., Lemaréchal H., Mir O., Borderie D., Tréluyer J.M. (2014). Treatment of oxaliplatin-induced peripheral neuropathy by intravenous mangafodipir. J. Clin. Investig..

[9] Karlsson J.O.G., Ignarro L.J., Lundström I., Jynge P., Almén T. (2015). Calmangafodipir [Ca4Mn(DPDP)5], mangafodipir (MnDPDP) and MnPLED with special reference to their SOD mimetic and therapeutic properties. Drug. Discov. Today.

[10] Schmidt P.P., Toft K.G., Skotland T., Andersson K. (2002). Stability and transmetallation of the magnetic resonance contrast agent MnDPDP measured by EPR. J. Biol. Inorg. Chem..

[11] Glimelius B., Manojlovic N., Pfeiffer P., Mosidze B., Kurteva G., Karlberg M., Mahalingam D., Buhl Jensen P., Kowalski J., Bengtson M. (2018). Persistent prevention of oxaliplatin-induced peripheral neuropathy using calmangafodipir (PledOx^®^): A placebo-controlled randomised phase II study (PLIANT). Acta Oncol..

[12] Karlsson J.O.G., Kurz T., Flechsig S., Näsström J., Andersson R.G. (2012). Superior therapeutic index of calmangafodipir in comparison to mangafodipir as a chemotherapy adjunct. Transl. Oncol..

[13] Lustberg M.B., Pfeiffer P., Qvortrup C., Mura K., Bengtson M.H., Nittve M., Sonesson C., Nagahama F., Sonehara Y., Carlsson C.S. (2019). The Global POLAR program: Two pivotal placebo-controlled studies of calmangafodipir used on top of modified FOLFOX6 to prevent chemotherapy-induced peripheral neuropathy (CIPN). J. Clin. Oncol..

[14] Renn C.L., Carozzi V.A., Rhee P., Gallop D., Dorsey S.G., Cavaletti G. (2011). Multimodal assessment of painful peripheral neuropathy induced by chronic oxaliplatin-based chemotherapy in mice. Mol. Pain.

[15] Marmiroli P., Riva B., Pozzi E., Ballarini E., Lim D., Chiorazzi A., Meregalli C., Distasi C., Renn C.L., Semperboni S. (2017). Susceptibility of different mouse strains to oxaliplatin peripheral neurotoxicity: Phenotypic and genotypic insights. PLoS ONE.

[16] Cavaletti G., Tredici G., Marmiroli P., Petruccioli M.G., Barajon I., Fabbrica D. (1992). Morphometric study of the sensory neuron and peripheral nerve changes induced by chronic cisplatin (DDP) administration in rats. Acta Neuropathol..

[17] Cavaletti G., Gilardini A., Canta A., Rigamonti L., Rodriguez-Menendez V., Ceresa C., Marmiroli P., Bossi M., Oggioni N., D’Incalci M. (2007). Bortezomib-induced peripheral neurotoxicity: A neurophysiological and pathological study in the rat. Exp. Neurol..

[18] Meregalli C., Fumagalli G., Alberti P., Canta A., Carozzi V.A., Chiorazzi A., Monza L., Pozzi E., Sandelius Å., Blennow K. (2018). Neurofilament light chain as disease biomarker in a rodent model of chemotherapy induced peripheral neuropathy. Exp. Neurol..

[19] Kerckhove N., Collin A., Condé S., Chaleteix C., Pezet D., Balayssac D. (2017). Long-Term Effects, Pathophysiological Mechanisms, and Risk Factors of Chemotherapy-Induced Peripheral Neuropathies: A Comprehensive Literature Review. Front. Pharmacol..

[20] Ewertz M., Qvortrup C., Eckhoff L. (2015). Chemotherapy-induced peripheral neuropathy in patients treated with taxanes and platinum derivatives. Acta Oncol..

[21] Marmiroli P., Cavaletti G. (2016). Drugs for the treatment of peripheral neuropathies. Expert. Opin. Pharmacol..

[22] Cavaletti G., Marmiroli P. (2015). Chemotherapy-induced peripheral neurotoxicity. Curr. Opin. Neurol..

[23] Di Cesare Mannelli L., Zanardelli M., Failli P., Ghelardini C. (2013). Oxaliplatin-induced oxidative stress in nervous system-derived cellular models: Could it correlate with in vivo neuropathy?. Free Radic. Biol. Med..

[24] Azevedo M.I., Pereira A.F., Nogueira R.B., Rolim F.E., Brito G.A., Wong D.V.T., Lima-Júnior R.C., De Albuquerque Ribeiro R., Vale M.L. (2013). The antioxidant effects of the flavonoids rutin and quercetin inhibit oxaliplatin-induced chronic painful peripheral neuropathy. Mol. Pain.

[25] Joseph E.K., Chen X., Bogen O., Levine J.D. (2008). Oxaliplatin acts on IB4-positive nociceptors to induce an oxidative stress-dependent acute painful peripheral neuropathy. J. Pain.

[26] Mangus L.M., Rao D.B., Ebenezer G.J. (2020). Intraepidermal Nerve Fiber Analysis in Human Patients and Animal Models of Peripheral Neuropathy: A Comparative Review. Toxicol. Pathol..

[27] Anand P., Elsafa E., Privitera R., Naidoo K., Yiangou Y., Donatien P., Gabra H., Wasan H., Kenny L., Rahemtulla A. (2019). Rational treatment of chemotherapy-induced peripheral neuropathy with capsaicin 8% patch: From pain relief towards disease modification. J. Pain Res..

[28] Cuzzocrea S., Riley D.P., Caputi A.P., Salvemini D. (2001). Antioxidant therapy: A new pharmacological approach in shock, inflammation, and ischemia/reperfusion injury. Pharmacol. Rev..

[29] Bonetta R. (2018). Potential Therapeutic Applications of MnSODs and SOD-Mimetics. Chemistry.

[30] McCord J.M. (2008). Superoxide dismutase, lipid peroxidation, and bell-shaped dose response curves. Dose Response.

[31] Mao G.D., Thomas P.D., Lopaschuk G.D., Poznansky M.J. (1993). Superoxide dismutase (SOD)-catalase conjugates. Role of hydrogen peroxide and the Fenton reaction in SOD toxicity. J. Biol. Chem..

[32] Towart R., Karlsson J.O.G., Jynge P. (1997). Reduction of Cardiotoxicity of an Antitumor Agent Using Manganese Compound. U.S. Patent.

[33] Archibald F.S., Fridovich I. (1982). The scavenging of superoxide radical by manganous complexes: In vitro. Arch. Biochem. Biophys..

[34] Yri O.E., Vig J., Hegstad E., Hovde Ø., Pignon I., Jynge P. (2009). Mangafodipir as a cytoprotective adjunct to chemotherapy—A case report. Acta Oncol..

[35] Iveson T.J., Kerr R.S., Saunders M.P., Cassidy J., Hollander N.H., Tabernero J., Haydon A., Glimelius B., Harkin A., Allan K. (2018). 3 versus 6 months of adjuvant oxaliplatin-fluoropyrimidine combination therapy for colorectal cancer (SCOT): An international, randomized, phase 3, non-inferiority trial. Lancet Oncol..

